# Risk of Incidence and Lethality by Etiology of Severe Acute Respiratory Syndrome in Hospitalized Children Under 1 Year of Age in Brazil in 2024: A Cross-Sectional Study

**DOI:** 10.3390/tropicalmed10060168

**Published:** 2025-06-14

**Authors:** Tamires de Nazaré Soares, Natasha Cristina Oliveira Andrade, Suziane do Socorro dos Santos, Marcela Raíssa Asevedo Dergan, Karina Faine Freitas Takeda, Jully Greyce Freitas de Paula Ramalho, Luany Rafaele da Conceição Cruz, Perla Katheleen Valente Corrêa, Marli de Oliveira Almeida, Joyce dos Santos Freitas, Wilker Alves Silva, Marcos Jessé Abrahão Silva, Daniele Melo Sardinha, Luana Nepomuceno Gondim Costa Lima

**Affiliations:** 1 Programa de Pós-Graduação em Biologia Parasitária na Amazônia, Universidade do Estado do Pará e Instituto Evandro Chagas (PPGBPA/UEPA/IEC), Belem 66087-662, Pará, Brazil; tamiresenfsoares@hotmail.com (T.d.N.S.); natasha.andrade88@hotmail.com (N.C.O.A.); susocorrosantos@gmail.com (S.d.S.d.S.); derganm20@gmail.com (M.R.A.D.); jesseabrahao10@gmail.com (M.J.A.S.); 2 Programa de Pós-Graduação em Enfermagem, Universidade do Federal do Pará (PPGENF/UFPA), Belem 66087-662, Pará, Brazil; karina.faine@gmail.com; 3 Programa de Pós-Graduação em Enfermagem, Universidade do Estado do Pará e Universidade Federal do Amazonas (PPGENF/UEPA-UFAM), Belem 66087-662, Pará, Brazil; jullygreyce@gmail.com; 4 Programa de Pós-Graduação em Virologia, Instituto Evandro Chagas (PPGV/IEC), Ananindeua 67000-000, Pará, Brazil; lu@luanycruz.com.br (L.R.d.C.C.); perlakvc@gmail.com (P.K.V.C.); 5 Programa de Pós-Graduação em Análises Clínicas, Universidade Federal do Pará (PPGAC/UFPA), Belem 66087-662, Pará, Brazil; marli.almeida@icb.ufpa.br; 6 Centro de Ciências Biológicas e da Saúde, Faculdade de Enfermagem, Universidade Federal do Pará (CCBS/FAENF/UFPA), Belem 66087-662, Pará, Brazil; joycefreitas@ufpa.br; 7 Programa de Pós-Graduação em Epidemiologia e Vigilância em Saúde, Instituto Evandro Chagas (PPGEVS/IEC), Ananindeua 67000-000, Pará, Brazil; wilkeralves18@gmail.com

**Keywords:** severe acute respiratory syndrome, children <1 year old, hospitalized, incidence, lethality, Brazil

## Abstract

Severe Acute Respiratory Syndrome (SARS) represents a significant cause of morbidity and mortality in children under one year of age, a particularly vulnerable population due to immunological and respiratory immaturity. The diverse etiology includes multiple respiratory viruses such as Respiratory Syncytial Virus (RSV), influenza, rhinovirus, and SARS-CoV-2, each with distinct potential to cause severe illness and death. Understanding the specific incidence and lethality by etiological agents in the recent Brazilian context (2024), after the COVID-19 pandemic, is essential to guide surveillance and public health strategies. This study aimed to analyze the risk of incidence and lethality by specific etiology of SARS in children under one year of age hospitalized in Brazil during the year 2024. A descriptive cross-sectional study was performed using secondary data from the 2024 Influenza Epidemiological Surveillance Information System (SIVEP-Gripe), obtained via OpenDataSUS. Reported cases of SARS hospitalized in children <1 year of age in Brazil were included. Distribution by final classification and epidemiological week (EW) was analyzed; the incidence rate by Federative Unit (FU) (cases/100,000 < 1 year) with risk classification (Low/Moderate/High) was assessed; and, for cases with positive viral RT-PCR, the etiological frequency and virus-specific lethality rate (deaths/total cases of etiology ×100), also with risk classification, were extracted. A multivariate logistic regression model was performed for the risk factors of death. A total of 66,170 cases of SARS were reported in children under 1 year old (national incidence: 2663/100,000), with a seasonal peak between April and May. The majority of cases were classified as “SARS due to another respiratory virus” (49.06%) or “unspecified” (37.46%). Among 36,009 cases with positive RT-PCR, RSV (50.06%) and rhinovirus (26.97%) were the most frequent. The overall lethality in RT-PCR-positive cases was 1.28%. Viruses such as parainfluenza 4 (8.57%), influenza B (2.86%), parainfluenza 3 (2.49%), and SARS-CoV-2 (2.47%) had higher lethality. The multivariate model identified parainfluenza 4 (OR = 6.806), chronic kidney disease (OR = 3.820), immunodeficiency (OR = 3.680), Down Syndrome (OR = 3.590), heart disease (OR = 3.129), neurological disease (OR = 2.250), low O_2_ saturation (OR = 1.758), SARS-CoV-2 (OR = 1.569) and respiratory distress (OR = 1.390) as risk factors for death. Cough (OR = 0.477) and RSV (OR = 0.736) were associated with a lower chance of death. The model had good calibration (Hosmer–Lemeshow *p* = 0.693) and overall significance (*p* < 0.001). SARS represented a substantial burden of hospitalizations, with marked seasonal and geographic patterns. RSV and rhinovirus were the main agents responsible for the volume of confirmed cases but had a relatively low to moderate risk of lethality. In contrast, less frequent viruses such as parainfluenza 4, influenza B, parainfluenza 3, and SARS-CoV-2 were associated with a significantly higher risk of death. These findings highlight the importance of dissociating frequency from lethality and reinforce the need to strengthen etiological surveillance, improve diagnosis, and direct preventive strategies (such as immunizations) considering the specific risk of each pathogen for this vulnerable population.

## 1. Introduction

Acute respiratory infections (ARIs) represent one of the leading causes of morbidity and mortality worldwide, disproportionately affecting vulnerable populations such as young children, the elderly, and individuals with comorbidities. The magnitude of the public health impact of Healthcare-associated infections (HAIs) is vast, overwhelming health systems and resulting in significant socioeconomic costs. Continuous epidemiological surveillance and understanding of the factors associated with the severity of these infections are therefore critical to the development of effective prevention and control strategies [1,2].

Within the spectrum of ARIs, Severe Acute Respiratory Syndrome (SARS) stands out as a clinical condition of high complexity and potential lethality [3]. Defined by the presence of severe respiratory signs and symptoms, often accompanied by hypoxemia and the need for ventilatory support, SARS requires hospitalization and intensive care in most cases [4]. Proper diagnosis and management are crucial, but the etiological heterogeneity of the syndrome poses a constant challenge to clinical practice and public health [5].

The pediatric population, in particular infants under one year of age, is a high-risk group for the development of severe forms of respiratory infections [6]. The immaturity of the immune system [7], the smaller airways and the lower physiological reserve [8] contribute to increased susceptibility to complications and unfavorable outcomes. Hospitalization for SARS in this age group is an important sentinel event, indicating the circulation of virulent respiratory pathogens and the need for targeted interventions [9].

The etiology of SARS is diverse, involving a wide range of infectious agents, especially respiratory viruses such as Respiratory Syncytial Virus (RSV) [10], influenza (A and B) [11], parainfluenza, adenovirus, human metapneumovirus [12] and, more recently, SARS-CoV-2 [13]. Bacteria, such as *Streptococcus pneumoniae* and *Haemophilus influenza*, can also cause or complicate SARS, although often as co-infections or secondary infections [14]. Accurate identification of the etiologic agent is crucial, as it influences prognosis, therapeutic options (when available), and infection control measures [15].

Understanding the risk associated with each specific etiology is vital. Different pathogens may have different virulence, transmissibility, and seasonality profiles [16], resulting in significant variations in the likelihood of causing severe disease and the likelihood of leading to death among severe cases [17]. As well as information on seasonality, it is crucial for planning health services [18,19].

The epidemiological scenario of respiratory infections is dynamic, influenced by factors such as the introduction of new pathogens, changes in population immunity (natural or vaccine-induced), climatic variations, and social behaviors [20,21]. The post-COVID-19 pandemic period, for example, brought changes in the circulation patterns of several respiratory viruses [22,23], reinforcing the need for continuous and up-to-date monitoring to inform health policies and outbreak preparedness [24].

Brazil, with its vast territorial extension and climatic and socioeconomic diversity [25], presents a complex landscape for SARS surveillance. The Influenza Epidemiological Surveillance System (SIVEP-Gripe), strengthened during the pandemic [26], has become an essential tool for monitoring hospitalized SARS cases across the country, allowing the collection of demographic, clinical, and laboratory data [27]. The analysis of these data is essential to understand the regional and national particularities of the syndrome, such as underreporting, investigation with gold standard sample collection, and outcome [28].

Focusing on the year 2024 allows us to capture the most recent epidemiology of SARS in infants in Brazil, reflecting the current patterns of viral circulation and the impact of current prevention and control strategies. Contemporary data are essential for timely decision-making, guiding resource allocation, vaccination campaigns such as influenza and, potentially, future RSV vaccines, and updated clinical protocols.

Despite the existence of surveillance systems, there are still gaps in detailed knowledge about the specific risk of SARS incidence and lethality attributable to each etiology in hospitalized infants in the recent Brazilian context. Studies that dissect these risks by etiological agents are needed to identify pathogens with the greatest impact on this vulnerable population and target prevention and treatment efforts more effectively. No studies in Brazil have been conducted in 2024 with hospitalized patients under 1 year of age. Evaluating the post-COVID-19 scenario allows us to understand seasonality and outcomes by etiology and prepare public health to cope with the coming seasons.

In this context, the main objective of the present study is to analyze the risk of incidence and lethality by specific etiology of SARS in children under one year of age hospitalized in Brazil during the year 2024. By investigating the contribution of different pathogens to the burden of severe and fatal SARS in this population, we seek to provide subsidies based on recent evidence for epidemiological surveillance, pediatric clinical practice, and the formulation of public health policies aimed at the protection of Brazilian infants.

## 2. Methodology

### 2.1. Study Design and Data Source

This is an epidemiological, descriptive, and cross-sectional study, using publicly accessible secondary data. The data were obtained from the database of the Influenza Epidemiological Surveillance Information System (SIVEP-Gripe), referring to the year 2024. SIVEP-Gripe is the official system of the Brazilian Ministry of Health for the registration and monitoring of cases of Severe Acute Respiratory Syndrome (SARS) hospitalized throughout the national territory. The anonymized microdata were accessed through the OpenDataSUS (https://opendatasus.saude.gov.br/dataset/?q=SARS) (Accessed 15 February 2025) platform.

### 2.2. Study Location

The present study covers the entire national territory of Brazil; a federative republic located in South America. With an area of more than 8.5 million square kilometers, it is the fifth largest country in the world by land area and the seventh most populous, with an estimated population of more than 215 million inhabitants (estimated for the study period). The country is administratively divided into 27 Federative Units (FUs)—26 states and the Federal District—grouped into five major geographic macro-regions (North, Northeast, Midwest, Southeast, and South) [29].

### 2.3. Study Population and Period

The study population comprised all reported cases of SARS in children under 1 year of age (0 to 11 months and 29 days) who were hospitalized in Brazil and whose records were included in the SIVEP-Gripe database during the 52 epidemiological weeks (EW) of 2024 (approximately from 31 December 2023, to 28 December 2024).

### 2.4. Case Definition and Variables Analyzed

The SARS case definition followed the criteria established by the Brazilian Ministry of Health, which generally include an individual hospitalized with fever (even if referred), cough OR sore throat AND dyspnea OR O^2^ saturation < 95% on room air OR respiratory distress [30].

The following variables were extracted and analyzed from the SIVEP-Gripe database: Age: Used to filter cases in children under 1 year of age. Notification Date: Used to determine the Epidemiological Week (EW) of the occurrence. Federative Unit (UF) of Residence: Used for analysis of geographic distribution and calculation of incidence. Final Case Classification: Variable used to describe the general distribution of cases according to the categories pre-defined in the system (SARS due to influenza, SARS due to another respiratory virus, SARS due to another etiological agent, SARS not specified, SARS due to COVID-19, not filled in). RT-PCR test result for respiratory viruses: To identify the specific viral etiology (influenza A, influenza B, Respiratory Syncytial Virus (RSV), parainfluenza 1, 2, 3, 4, adenovirus, metapneumovirus, bocavirus, rhinovirus, SARS-CoV-2, other respiratory virus). Evolution of the case was included to assess the outcome.

The RT-PCR was carried out by the State Central Laboratories (LACEN), using the CDC Protocol-Respiratory Viruses (CDC/Atlanta/USA). The analysis for the new coronavirus (SARS-CoV-2) was carried out using the CDC/USA Protocol: SARS-CoV-2 (N1 and N2) as recommended by the World Health Organization and the Ministry of Health, with the reagent kit for detecting SARS-CoV-2 IDT. The method used to detect SARS-CoV-2 is real-time RT-PCR using the protocols provided by the CDC (Centers for Disease Control) or the Charité Institute of Virology–University of Berlin.

### 2.5. Data Processing and Analysis

Data were downloaded from the OpenDataSUS platform and processed using the Statistical Package for the Social Sciences (SPSS) 25 statistical software (https://www.ibm.com/br-pt/spss/) (Accessed 15 February 2025). The electronic spreadsheets were processed in Microsoft Excel 2019.

In the general descriptive analysis, we calculated the absolute (N) and relative (%) frequencies of SARS cases hospitalized in children under 1 year of age according to the “Final Classification” variable, Influenza, Another respiratory virus, Another etiologic agent, Unspecified, COVID-19, Not filled.

In the temporal analysis, the reported cases were aggregated by Epidemiological Week (EW) of occurrence to visualize the temporal distribution and seasonality throughout the year 2024, presented in a graph.

The incidence rate of hospitalized SARS per FU was calculated by dividing the total number of cases reported in children under 1 year of age living in each FU by the total estimated population for this age group (under 1 year of age) in the respective FU in 2024, multiplied by 100,000 inhabitants. Population estimates for children under 1 year of age per state for 2024 were obtained from DataSUS (http://tabnet.datasus.gov.br/cgi/tabcgi.exe?ibge/cnv/popsvs2024br.def) (Accessed 27 February 2025).

We analyzed the percentage of RT-PCR-positive cases by state, showing the percentage of positive cases for each state using a map made in Excel 2019.

FUs were classified into incidence risk categories (Low, Moderate, High) based on comparing their incidence rate with the national average rate (2663 cases/100,000). The cut-off points used were as follows: Low (<1500/100,000), Moderate (≥1500 and ≤4000/100,000), and High (>4000/100,000) as shown in the table.

In the etiological and lethality analysis, only SARS cases in children under 1 year of age who had a respiratory sample collected and tested positive in the RT-PCR test for at least one respiratory virus were selected for analysis. Cases with closure by other criteria (antigen test, serology, clinical–epidemiological) or with negative/not performed RT-PCR were excluded from this specific sub-analysis. For this subgroup, we calculated the absolute (N) and relative (%) frequencies of each identified viral etiology. We calculated the number of survivors and deaths for each etiology. We calculated the specific lethality rate for each viral etiology by dividing the number of deaths by the total number of cases of that etiology, multiplied by 100 (number of deaths/total chaos ×100). The overall case fatality rate for this subgroup (positive RT-PCR) was also calculated.

We classified the “Risk of Lethality” by etiology by comparing the specific lethality rate with the overall average lethality rate (1.28%). The cut-off points used were: Low (<1.15%), Moderate (≥1.15% and ≤2.0%), and High (>2.0%), as shown in the table.

A multivariate binary logistic regression model was used to identify the factors associated with death in children under 1 year of age hospitalized for SARS in Brazil in 2024. The dependent variable (outcome) was death, coded dichotomously (e.g., 1 for death, 0 for survival/discharge).

The independent variables (predictors) included in the final multivariate model were gender, signs and symptoms, risk factors, and comorbidity and etiology. These variables were treated as dichotomous categorical (presence (1) vs. absence of the factor or condition (0)).

The multivariate model was built to estimate the adjusted Odds Ratios (OR) and their respective 95% Confidence Intervals (95% CI) for each predictor variable, simultaneously controlling for the effect of the other variables included in the model. The statistical significance of the associations was assessed using the *p*-value, considering a significance level of α = 0.05.

The assessment of the quality and fit of the final multivariate model included the following steps and metrics: Overall significance of the model: Verified by the Omnibus Test of Model Coefficients. A *p*-value < 0.05 in this test indicates that the model with the predictor variables is statistically superior to a null model (with only the intercept), i.e., that the variables included together contribute significantly to explaining the outcome. Model calibration: Assessed using the Hosmer-Lemeshow test. This test compares the observed death frequencies with the frequencies predicted by the model in different risk deciles. A *p*-value > 0.05 in this test suggests that there is no statistically significant difference between the observed and predicted frequencies, indicating good model calibration.

### 2.6. Ethical Considerations

The study used exclusively secondary, anonymous data in the public domain, made available by the Ministry of Health through the OpenDataSUS platform. According to Resolution No. 510/2016 of the National Health Council (CNS) of Brazil, research that uses publicly accessible information, without identifying the participants, is exempt from consideration by the Research Ethics Committee (CEP) [31]. All procedures ensured the confidentiality and anonymity of the individuals included in the original database.

## 3. Results

In the period analyzed in 2024, a total of 66,170 cases of SARS in children under 1 year of age were reported and hospitalized in Brazil. The distribution of these cases according to the final classification was as follows: SARS caused by another respiratory virus was the most frequent classification, corresponding to 32,466 cases, which represents (49.06%) of the total. Unspecified SARS (did not collect a sample or had a negative RT-PCR or antigen result), the second largest category, with 24,790 cases, was equivalent to (37.46%) of the total. SARS due to COVID-19, 3342 cases were registered, corresponding to (5.05%) of the total. Influenza represented 2560 cases, or (3.87%) of the total. There were 2271 cases not filled, which is equivalent to (3.43%) of the total. Another etiological agent (bacterial or fungal causes) was the least frequent classification among the specified causes, with 741 cases, representing 1.12% of the total (Table 1).

Most hospitalizations for SARS in children under 1 year of age in 2024 were classified as caused by other respiratory viruses or remained with the cause unspecified. COVID-19 and influenza accounted for smaller, but still significant, proportions of cases.

The graph details the number of SARS cases reported weekly in children under 1 year of age over the 52 epidemiological weeks of the year. The data show a clear seasonal variation.

At the beginning of the year (EW 1 to EW 8), there was a relatively low number of notifications. EW 1 recorded 468 cases. In the following weeks, the numbers fluctuated, with the lowest value in this period being 384 cases in EW 3 and the highest 590 cases in EW 8. Cases generally remained below 500 per week until EW 7. The period of increase (EW 9 to EW 17), from EW 9 (679 cases), shows a significant and continuous weekly increase. Cases exceeded 1000 in EW 11 (1107), and 2000 in EW 15 (2336), culminating in the peak in EW 17. The seasonal peak (EW 17 to EW 21) was concentrated between weeks 17 and 21. The absolute peak occurred in EW 17, with 2742 cases reported. The subsequent weeks also recorded very high numbers: EW 18 (2557), EW 19 (2670), EW 20 (2640) and EW 21 (2499). Even weeks 22 to 25 remained above 2000 cases (with a secondary peak in EW 23 with 2440 cases).

The period of decline (EW 22 to EW 33) followed EW 21 (or EW 23, considering the secondary peak), and a general downward trend began. Notifications fell below 2000 cases in EW 26 (1947) and below 1600 in EW 28 (1512). The drop continued to reach 1023 cases in EW 33. At the end of the year (EW 34 to EW 52), in the last part of the year, the number of weekly cases stabilized at a moderate level, with fluctuations. The values ranged mainly from 744 (EW 46) to 1192 (EW 34). There was a notable drop in the last week of the year, EW 52, which recorded 560 cases.

**Figure 1:**
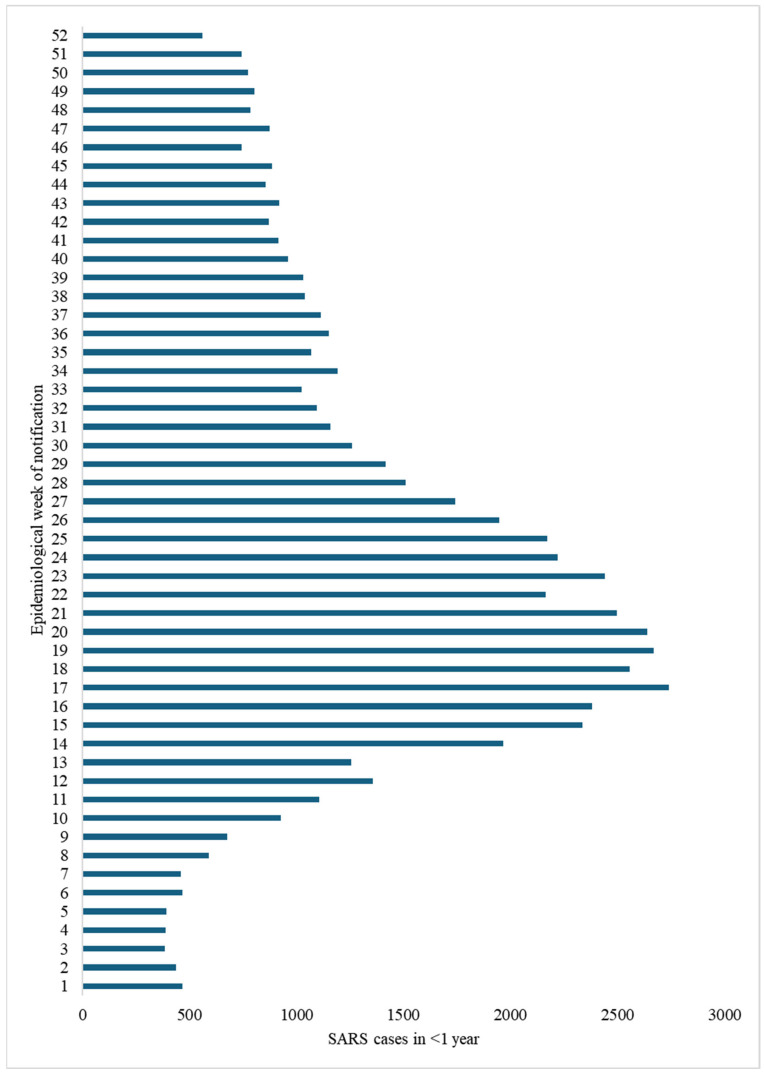
Notifications per epidemiological week of SARS in children under 1 year of age who were hospitalized in Brazil in 2024. Source: OpenDataSUS.

SARS reporting in children under 1 year of age exhibited strong seasonality, starting at low levels, growing rapidly to a sharp peak between weeks 17 and 21 (roughly corresponding to April/May), followed by a decline and stabilization at moderate levels (between 750 and 1200 cases/week) during the second half of the year, with a final drop in the last week. The highest number of weekly cases was 2742 (EW 17) and the lowest was 384 (EW 3) (Figure 1).

Table 2 presents the number of SARS cases in children under one year of age, the estimated population in this age group, and the incidence rate (cases per 100,000 inhabitants < 1 year) for each Federative Unit (FU) of Brazil in 2024. The average national incidence was 2663 cases per 100,000 children under 1 year of age. An additional column classifies the “Incidence Risk” as Low, Moderate, or High, comparing the incidence of each FU to the national average.

There is a wide variation in incidence among FUs, ranging from 371/100,000 in Piauí (Low Risk) to 7301/100,000 in the Federal District (High Risk). States with Low Incidence Risk (<1500/100,000) were Alagoas, Maranhão, Mato Grosso, Pará and Piauí. States with Moderate Incidence Risk (~1500 to 4000/100,000), including most FUs and those with the highest absolute numbers of cases (such as São Paulo, Minas Gerais, Rio de Janeiro, Paraná, Rio Grande do Sul) were the following: Amazonas, Bahia, Ceará, Espírito Santo, Goiás, Paraíba, Pernambuco, Paraná, Rio de Janeiro, Rio Grande do Norte, Roraima, Rondônia, Rio Grande do Sul, São Paulo, Tocantins. States with High Incidence Risk (>4000/100,000) were Acre, Amapá, Federal District, Mato Grosso do Sul, Santa Catarina and Sergipe (Table 2).

This table does not assess the severity of the disease (lethality), but rather the risk of occurrence of a case of SARS requiring hospitalization among the population of children under one year of age in each state. States classified as “High Risk” are those where SARS had the greatest proportional impact on the child population in 2024, suggesting greater viral circulation, greater vulnerability of the local population, or potentially greater detection and reporting capacity. States with “Low Risk” had a proportionally lower burden of the disease. “Moderate Risk” states, even if they include those with a large volume of cases (such as SP, MG), had an occurrence rate per inhabitant closer to the national average. Categorization helps to geographically identify the areas with the greatest pressure from SARS on the child population and, consequently, on pediatric health services. The reasons for these differences in incidence can be multifactorial (climatic and socioeconomic factors, population density, access to healthcare, specific circulation of viruses, underreporting SARS, etc.) (Table 2).

Looking at the percentage of positive cases of respiratory viruses (detected by RT-PCR) in samples from children under the age of 1 hospitalized for Severe Acute Respiratory Syndrome (SARS) by Federative Unit in Brazil in 2024, there is considerable variation. At the top of the list, Santa Catarina recorded the highest rate, with 70.19% positivity, followed very closely by Mato Grosso, with 70.17%. The Federal District also had a very high percentage, at 68.58%. With rates above 60%, but below the leaders, is Rondônia with 64.57%. A significant group of states had percentages in the 50% range: Bahia (59.98%), Goiás (58.97%), Paraná (55.62%), Rio Grande do Norte (55.26%), Acre (53.92%), Roraima (51.96%) and Rio Grande do Sul (50.89%). In the 40% range are Espírito Santo (49.25%), Amazonas (47.93%), Amapá (47.83%), Alagoas (47.06%), Paraíba (45.04%), Minas Gerais (44.60%), Rio de Janeiro (43.56%) and Sergipe (42.97%). With rates below 40%, but still above 30%, were Tocantins (36.86%), São Paulo (34.72%), Pará (34.00%), and Maranhão (33.83%). The lowest percentages of positivity were observed in Pernambuco (26.97%), Piauí (15.69%), and, notably, Mato Grosso do Sul, with just 9.12%.

**Figure 2:**
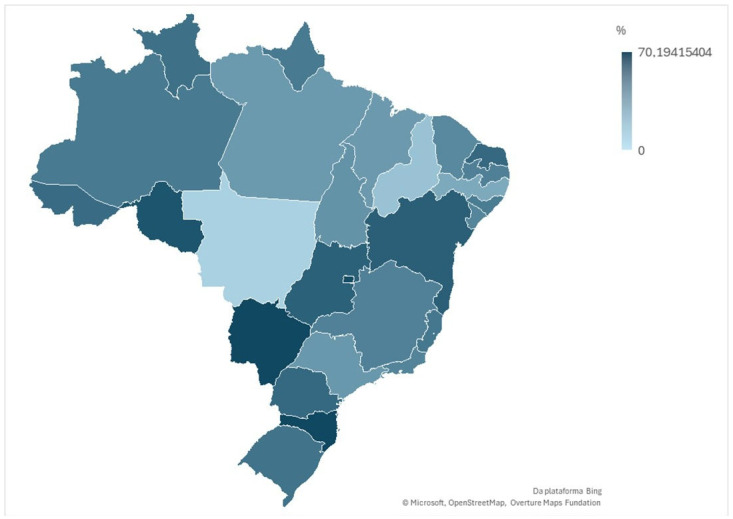
Percentage of positive cases sampled for RT-PCR for respiratory viruses in children under 1 hospitalized for SARS by the federative unit in Brazil in 2024. Source: OpenDataSUS.

In comparison, the disparity is stark. States such as Santa Catarina and Mato Grosso presented a scenario where more than 7 out of every 10 children tested in this condition tested positive for respiratory viruses. In contrast, in Mato Grosso do Sul, less than 1 in 10 children tested positive. Most states were between 40% and 60% positive, but the extremes demonstrate the heterogeneity of the situation in the country (Figure 2).

When we filtered for cases that collected samples for the gold standard test, RT-PCR, and were positive for a respiratory virus, we analyzed 36,009 hospitalizations for SARS in children under one year of age. Cases confirmed by rapid antigen tests, serology, and clinical and epidemiological tests were excluded from the analysis. Of this total, the vast majority—35,548 patients (98.72%)—survived hospitalization, while 461 patients (1.28%) died. The overall lethality rate for SARS in this age group was, therefore, 1.28% (Table 3).

The main cause of hospitalization was Respiratory Syncytial Virus (RSV), responsible for 18,026 cases, which represents (50.06%) of the total, that is, more than half of all hospitalizations due to SARS in this group. Of these RSV cases, 17,841 survived and 185 died. The second most frequent etiological agent was rhinovirus, with 9710 cases (26.97%). For this etiology, 9570 survivors and 140 deaths were recorded. Next, we have SARS-CoV-2, with 2144 hospitalizations (5.95%), of which 2091 resulted in discharge and 53 in death. Adenovirus was responsible for 1731 cases (4.81%), with 1712 survivors and 19 deaths. Influenza A caused 1408 hospitalizations (3.91%), with 1390 survivors and 18 deaths. Metapneumovirus was identified in 1114 cases (3.09%), with 1104 survivors and 10 deaths (Table 3).

Other less frequent etiologies included the following: Parainfluenza 3 with 603 cases (1.67%), with 588 survivors and 15 deaths; the “Other virus” category with 515 cases (1.43%), with 509 survivors and 6 deaths; bocavirus with 362 cases (1.01%), with 358 survivors and 4 deaths; influenza B with 245 cases (0.68%), with 238 survivors and 7 deaths; parainfluenza 1 with 65 cases (0.18%), with 64 survivors and 1 death; parainfluenza 2 with 51 cases (0.14%), all survivors (0 deaths); and parainfluenza 4 with 35 cases (0.10%), with 32 survivors and 3 deaths (Table 3).

In the comparison of survivors vs. deaths, when comparing the proportion of deaths between the different etiologies (lethality rate), we observed important variations. The overall lethality was 1.28%. Respiratory Syncytial Virus (RSV), despite being the most common cause of hospitalization and responsible for the highest absolute number of deaths (185), had a lethality rate slightly lower than the average: 1.03% (185/18,026). Rhinovirus, the second most common and second in absolute number of deaths (140), had a lethality slightly above average: 1.44% (140/9710) (Table 3).

Agents with notably higher than average lethality included parainfluenza 4: 8.57% (3/35)—the highest, although based on few cases, influenza B: 2.86% (7/245), followed by parainfluenza 3: 2.49% (15/603) and SARS-CoV-2: 2.47% (53/2144) (Table 3).

Agents with lethality close to or below the average included parainfluenza 1: 1.54% (1/65), influenza A: 1.28% (18/1408)—exactly average, “Another virus”: 1.17% (6/515), adenovirus: 1.10% (19/1731), bocavirus: 1.10% (4/362), and metapneumovirus: 0.90% (10/1114). Parainfluenza 2 stood out for not having recorded any deaths (0% lethality) among the identified cases (Table 3).

In summary, RSV and rhinovirus are the main agents responsible for the volume of hospitalizations due to SARS in children under one year of age. However, the severity, measured by the lethality rate, is higher for infections by parainfluenza 4, influenza B, parainfluenza 3, and SARS-CoV-2 in this age group, indicating the highest proportion of deaths for children hospitalized with these specific etiologies, compared to the general average or viruses such as RSV and metapneumovirus. The high number of deaths from RSV and rhinovirus is due more to their high incidence than to an intrinsically high lethality.

Regarding the risk of incidence, the Low-Risk category (<1.15% lethality) includes RSV (1.03%), parainfluenza 2 (0.00%), adenovirus (1.10%), metapneumovirus (0.90%) and bocavirus (1.10%). Notably, parainfluenza 2 had no deaths recorded. The Moderate Risk category (close to average case fatality, ~1.15% to 2.0%) includes influenza A (1.28%), parainfluenza 1 (1.54%), rhinovirus (1.44%), and “Other virus” (1.17%). The High-Risk category (>2.0% lethality) includes influenza B (2.86%), parainfluenza 3 (2.49%), parainfluenza 4 (8.57%) and SARS-CoV-2 (2.47%). Parainfluenza 4 had the highest lethality, although with few cases (Table 3).

The table reveals that the frequency of a virus as a cause of hospitalization does not directly correlate with its risk of leading to death. RSV, although it causes most hospitalizations and the highest absolute number of deaths (185), has a relative lethality risk classified as low. In contrast, less common viruses such as influenza B, parainfluenza 3, parainfluenza 4, and SARS-CoV-2, when they cause SARS in children under one year of age, are associated with a significantly higher risk of fatal outcome (High Risk). Rhinovirus and influenza A, although common, have a moderate risk of lethality, close to the general average. Risk categorization helps to quickly identify the viral agents that pose the greatest mortality danger for this vulnerable age group, regardless of their overall prevalence.

In the multivariate model, we found that the factors associated with death were as follows: Parainfluenza 4 by PCR was the risk factor of greatest magnitude, increasing the odds of death 6806 times (OR = 6806; 95% CI: 2052–22,572; *p* = 0.002). Chronic kidney disease was associated with a 3820-fold increase in the odds of death (OR = 3820; 95% CI: 1334–10,936; *p* = 0.013). Immunodeficiency increased the odds of death by 3680 times (OR = 3680; 95% CI: 1849–7322; *p* < 0.001). Down syndrome increased the odds of death by 3590 times (OR = 3590; 95% CI: 2167–5950; *p* < 0.001). Chronic heart disease as a comorbidity increased the odds of death by 3129 times (OR = 3129; 95% CI: 2069–4731; *p* < 0.001). Chronic neurological disease increased the odds of death by 2250 times (OR = 2250; 95% CI: 1275–3969; *p* = 0.005). Low oxygen saturation was associated with 1758-fold higher odds of death (OR = 1758; 95% CI: 1401–2205; *p* < 0.001). SARS-CoV-2 increased the odds of death by 1569 times (OR = 1569; 95% CI: 1144–2152; *p* = 0.005). Respiratory distress increased the odds of death by 1390 times (OR = 1390; 95% CI: 1041–1856; *p* = 0.026) (Table 4).

The factors associated with survival were as follows: RSV was associated with a lower chance of death, with 0.736 times the odds of death compared to those without detected RSV (OR = 0.736; 95% CI: 0.595–0.909; *p* = 0.005), a reduction of about 26.4% in the odds. Cough was associated with significantly lower odds of death, corresponding to 0.477 times the odds of those without cough (Odds Ratio [OR] = 0.477; 95% Confidence Interval [95% CI]: 0.377–0.604; *p* < 0.001). This represents a reduction of approximately 52.3% in the odds of death (Table 4).

In the multivariate model quality assessment, significance, by the Omnibus test, indicated that the model as a whole is statistically significant (Chi-square = 235,211, df = 11, *p* < 0.001). This means that the set of predictor variables included in the model significantly improves the ability to predict death compared to a model without predictors (only with the intercept). The model calibration, by the Hosmer–Lemeshow test, resulted in a Chi-square of 3882 with six degrees of freedom and a significance value (*p*) of 0.693. Since this *p*-value is greater than 0.05, there is no statistical evidence to reject the null hypothesis that the model is well-calibrated. This indicates a good fit between the death probabilities predicted by the model and the observed death rates in the different risk groups (Table 4).

## 4. Discussion

Our study provides a comprehensive overview of severe acute respiratory syndrome (SARS) in children under one year of age in Brazil in 2024, highlighting the magnitude of the problem with more than 66,000 reported hospitalizations. This number underlines the vulnerability of this age group to severe respiratory infections and the substantial impact they represent on the Brazilian pediatric health system, requiring an in-depth analysis of their causes, temporal patterns, and geographic distribution.

A meta-analysis showed that during the COVID-19 pandemic, enteroviruses/rhinoviruses and RSV were the most prevalent respiratory viruses among children. It also showed differences in regional, seasonal, and age group distribution. The highest prevalence was found in children up to 1 year of age. RSV prevalence increased in the second half of the pandemic [32].

RSV was the main cause of hospitalizations in children under 1 year of age in our study, among the positive cases by RT-PCR. An observational study in northwestern Italy analyzed the epidemiology and clinical course of bronchiolitis in 647 children aged 0 to 2 years hospitalized over a 5 year period, focusing on the impact of the COVID-19 pandemic. There was a significant increase in RSV detection during the pandemic years (from 51.5% to 74.5%) and a progressive increase in the number of children requiring respiratory support, RSV infections, and a history of prematurity over the period. However, there was no corresponding increase in the need for mechanical ventilation, duration of respiratory support, ICU admission, or length of hospital stay. The pandemic has significantly altered the epidemiology of bronchiolitis, especially in the 2021–2022 season, reinforcing the importance of RSV surveillance for the planning of prophylactic strategies and the preparation of health systems [33].

A survey at a children’s hospital in Italy showed that during the study period, there were 72,959 admissions to the emergency room for Acute Respiratory Failure (ARI) and 33,355 respiratory samples tested positive for the virus. Before the pandemic, RSV and influenza had a clear seasonal pattern, which was interrupted in 2020. In 2021–2022, RSV peaked at the highest level observed during the study period, while influenza activity was minimal. The peaks of emergency room visits for ARI corresponded to the peaks of influenza, RSV, and rhinovirus in the 2018–2019 and 2019–2020 seasons, SARS-CoV-2 and rhinovirus in 2020, and RSV and hPVI in 2021–2022. ARI that results in emergency department care or hospitalizations should be included in the measurement of the burden of disease by ARI for a more accurate measurement of the impact of preventive measures [34].

In the case of the fatality rate of hospitalized young children, the mean case fatality rate was 1.2% (range 0 to 8.3%; median 0%; n = 10) among preterm infants and 5.2% (range 2.0 to 37.0%; median 5.9%; n = 7) among children with congenital heart disease. Estimates of lethality among children without high risk (n = 6) ranged from 0% to 1.5% (weighted mean 0.2%; median 0.0%). Lethality during hospitalization for severe RSV Lower Respiratory Tract Infection (LRTI) is rare among children who are not at high risk but occurs more commonly among children at higher risk for RSV LRTI [35]. A meta-analysis highlighted that the main risk factor for death at <5 years with RSV is prematurity <37 weeks, with a risk of 3.81 (95% CI, 1.68–8.63) [36].

A study in Brazil from February 2020 to February 2023 showed that among children <2 years old hospitalized with acute respiratory infection, infection with coronavirus 2 SARS had the highest risk of in-hospital mortality compared to seasonal viruses. The presence of underlying medical conditions was a risk factor for death for all viruses [37]. In Brazil, another study at the beginning of the COVID-19 pandemic evaluated pediatric hospitalizations for SARS. The mortality rate was higher for COVID-19 (15.2% vs. 4.5% vs. 3.2%, *p* < 0.001), with a risk of death more than three times higher than the other respiratory viruses, RSV and influenza. The presence of two or more comorbidities further increased this risk (Odds Ratio [OR] = 4.8 [95% CI 3.5–6.6]). Severe COVID-19 had a higher mortality rate than other viral respiratory diseases, despite the lower frequency of fever, cough, dyspnea, respiratory distress, and desaturation. The risk of death was strongly associated with preexisting comorbidities [38].

A meta-analysis showed that hospitalized children and adolescents who are most vulnerable to severe disease or death from SARS-CoV-2 infection are infants <1 year, adolescents, people with heart or neurological problems, two or more comorbidities, and obese people. These groups should be considered of higher priority for vaccination and for the use of protective shielding, where appropriate [39].

In our study, the higher risk of mortality was associated with Human Parainfluenza Viruses (hPIV) type 4 and 3. A global burden meta-analysis estimated that in 2018 approximately 18.8 million (uncertainty interval [RH] 12.8–28.9) cases of hPIV, 725,000 (RH 433,000–1,260,000) hospital admissions, and 34,400 (RH 16,400–73,800) deaths were attributable to hPIV globally among children under 5 years of age. When analyzed in the context of estimates of the all-cause burden of LRTI, hPIV accounts for 13% of LRTI cases, 4% to 14% of LTRI hospital admissions, and 4% of LRTI mortality. Similar to other respiratory viruses, a higher proportion of hospital admissions for hPIV (61%) and hospital deaths (66%) occurred in infants under 1 year of age [40]. This explains the high lethality due to hPVI in our study of children under 1 year of age. The pediatric hospitalization burden for hPVI is substantial and highlights the need to find an effective vaccine candidate [41].

Regarding influenza, we showed that it presented a high risk of lethality for B and moderate for A. In a study from 2010 to 2016 in the United States of America (USA), there were 675 deaths from influenza. The mean age was 6 years. The mean annual incidence was 0.15 per 100,000 children and was highest among children aged < 6 months, followed by children aged 6–23 months. Only 31% of children aged ≥ 6 months had received any flu vaccination. Half of the children had no preexisting medical conditions. Compared to children with preexisting medical conditions, children without any were younger (median: 5 vs. 8 years), less vaccinated (27% vs. 36%), more likely to die before hospital admission (77% vs. 48%), and had a shorter duration of illness (4 vs. 7 days; *p* < 0.05 for all) [42]. In our study, we did not evaluate influenza vaccination, although lethality was similar.

Rhinovirus presented a risk of moderate lethality in our study. A cohort study followed 263 infants during the first year of life to investigate the role of common respiratory viruses in acute respiratory infections (ARIs). The babies had an average of 4.1 ARIs, with viruses detected in 69% of cases, with rhinovirus (48.5%) being much more frequent than RSV (10.9%). Although RSV is known to cause serious infections, the study revealed that rhinovirus was responsible for a significantly higher number of upper (>10×) and lower (>3×) respiratory tract infections, including those with wheezing, compared to RSV, establishing rhinovirus as the main overall respiratory pathogen in the first year of life, a previously underestimated role for lower tract infections [43]. In our study, rhinovirus was the second most prevalent etiology, while the first was RSV; however, it is worth noting that Brazil experienced an RSV epidemic in 2024 [44].

Therefore, the Brazilian Ministry of Health published a statement on 17 February 2025 to the effect that it will incorporate two new technologies into the Unified Health System (SUS) to combat serious complications from RSV in babies, following the recommendation of Conitec: a vaccine for pregnant women, which will protect the newborn through maternal antibodies, and the monoclonal antibody nirsevimab, intended for premature infants and children with comorbidities up to two years old. The measure aims to significantly reduce hospitalizations and infant mortality due to RSV, the main cause of bronchiolitis and pneumonia in children under two years of age, hoping to prevent about 28 thousand hospitalizations annually and expand protection to approximately 2 million babies (vaccine) and 300 thousand additional children (nirsevimab) compared to previous therapy (palivizumab), representing an advance in public health and childhood immunization in Brazil [45].

It is essential to recognize the limitations of this study. The large proportion of “unspecified” cases in the final classification is a significant limitation for complete etiological surveillance. The lethality analysis based only on cases with positive RT-PCR may not represent the totality of SARS cases, excluding those diagnosed by other methods or that did not have a sample collected/processed. In addition, the quality and completeness of OpenDataSUS data may vary. However, the strength lies in the large volume of national data, allowing robust analyses of seasonality, geographic incidence, and, in a substantial subgroup, of the detailed viral etiology and associated lethality.

The 2024 Brazilian data on SARS in children under 1 year of age confirm the enormous burden of this syndrome, with etiological (RSV and rhinovirus dominance in confirmed cases) and seasonal (autumn/winter peak) patterns largely consistent with the international literature, especially for the Southern Hemisphere. However, the high national incidence with extreme geographic variability and the dissociation between viral frequency and lethality risk stand out, with less common agents such as influenza B, parainfluenza 3–4, and SARS-CoV-2 presenting a higher risk of death. These findings have direct implications for public health, reinforcing the need to strengthen etiological surveillance (improve diagnosis and reduce unspecified cases), optimize the allocation of hospital resources during seasonal peaks, direct prevention strategies (such as influenza vaccination and future RSV and COVID-19 immunizations for pregnant women/infants), and investigate the factors that drive high incidence rates and regional disparities in Brazil.

## 5. Conclusions

Analysis of hospitalizations for Severe Acute Respiratory Syndrome (SARS)in infants under one year of age in Brazil during 2024 reveals a significant disease burden, with 66,170 reported cases and a national incidence of 2663 per 100,000 children in this age group. The data demonstrate strong seasonality, with peak occurrence concentrated between epidemiological weeks 17 and 21 (approximately corresponding to April/May), and a marked geographical variation in incidence among the Federative Units, indicating higher-risk areas such as the Federal District, Acre, Amapá, Mato Grosso do Sul, Santa Catarina, and Sergipe. Although “other respiratory viruses” are the most commonly recorded cause and a large proportion remains unspecified, detailed analysis of cases with viral diagnosis by RT-PCR confirms RSV (50.06%) and rhinovirus (26.97%) as the primary etiological agents responsible for the volume of hospitalizations.

Crucially, the study distinguishes frequency from case fatality rate: although RSV and rhinovirus cause the highest absolute number of hospitalizations and deaths (due to high incidence), their case fatality rates (1.03% and 1.44%, respectively) are classified as low to moderate. In contrast, less frequent viruses such as parainfluenza 4 (8.57%), influenza B (2.86%), parainfluenza 3 (2.49%), and SARS-CoV-2 (2.47%) present a significantly higher risk of death when they cause SARSin this vulnerable population. Therefore, SARS in infants in Brazil is a substantial public health problem with defined seasonal and geographical patterns, and its impact involves both high-prevalence, lower-lethality viruses (RSV, rhinovirus) and less common but more lethal viruses per infection.

Independent risk factors for death included the detection of parainfluenza 4, which emerged as the risk factor of greatest magnitude, followed by severe comorbidities such as chronic kidney disease, immunodeficiency, Down syndrome, and chronic heart disease. Signs of clinical severity, such as low oxygen saturation, respiratory distress, and SARS-CoV-2 infection also proved to be significant predictors of mortality. Interestingly, the presence of cough and RSV detection were associated with a lower chance of death in the adjusted model, suggesting that, when other factors are controlled for, these may be associated with less severe cases or different patient profiles.

The results indicate that while RSV and rhinovirus drive the volume of SARI hospitalizations in infants under 1 year, the severity and risk of death are substantially higher in children with certain pre-existing comorbidities or those infected by specific viruses such as parainfluenza 4 and SARS-CoV-2. The identification of these risk factors, including the high case fatality rate of less frequent viruses, is crucial for epidemiological surveillance, guiding preventive measures (where applicable), and optimizing the clinical management of pediatric SARI patients, aiming to reduce mortality in this vulnerable population.

## Figures and Tables

**Table 1:** SARS cases by final classification in children under 1 year of age who were hospitalized in Brazil in 2024.

Final Classification	N	%
**Influenza**	2560	3.87
**Another respiratory virus**	32,466	49.06
**Another etiologic agent**	741	1.12
**Unspecified**	24,790	37.46
**COVID-19**	3342	5.05
**Not filled**	2271	3.43
**Total**	66,170	100.00

Source: OpenDataSUS.

**Table 2:** SARS cases, risk and incidence by the federative unit in children under 1 year of age who were hospitalized in Brazil in 2024.

Federative Unit	SARS Cases	Population < 1	Incidence	Risk of Incidence
**Acre**	599	14,021	4272	High
**Alagoas**	374	45,026	831	Low
**Amazon**	1158	69,395	1669	Moderate
**Amapá**	692	13,004	5321	High
**Bahia**	3441	165,718	2076	Moderate
**Ceará**	2772	109,754	2526	Moderate
**Federal District**	2527	34,610	7301	High
**Espírito Santo**	1328	51,052	2601	Moderate
**Goias**	1974	89,695	2201	Moderate
**Maranhao**	881	95,397	924	Low
**Minas Gerais**	5870	229,106	2562	Moderate
**Mato Grosso do Sul**	1743	39,414	4422	High
**Mato Grosso**	362	57,903	625	Low
**Pará**	994	123,569	804	Low
**Paraíba**	1241	50,336	2465	Moderate
**Pernambuco**	2521	113,753	2216	Moderate
**Piaui**	153	41,251	371	Low
**Paraná**	4682	136,611	3427	Moderate
**Rio de Janeiro**	5248	172,985	3034	Moderate
**Rio Grande do Norte**	780	38,852	2008	Moderate
**Roraima**	358	13,335	2685	Moderate
**Rondônia**	477	23,398	2039	Moderate
**Rio Grande do Sul**	4270	117,703	3628	Moderate
**Santa Catarina**	4687	96,335	4865	High
**Sergipe**	1273	27,912	4561	High
**São Paulo**	15,358	491,712	3123	Moderate
**Tocantins**	407	22,497	1809	Moderate
**Brazil**	**66,170**	**2,484.344**	**2663**	

Source: OpenDataSUS.

**Table 3:** Etiology, outcome, and risk of lethality of SARS in children under 1 year of age who were hospitalized in Brazil in 2024.

Etiology	General	%	Survivor	Survivor Rate	Death	Case Fatality Rate %	Risk of Lethality
**Influenza A**	1408	3.91	1390	98.72	18	1.28	Moderate
**Influenza B**	245	0.68	238	97.14	7	2.86	High
RSV *	18,026	50.06	17,841	98.97	185	1.03	Low
**Parainfluenza 1**	65	0.18	64	98.46	1	1.54	Moderate
**Parainfluenza 2**	51	0.14	51	100,00	0	0.00	Low
**Parainfluenza 3**	603	1.67	588	97.51	15	2.49	High
**Parainfluenza 4**	35	0.10	32	91.43	3	8.57	High
**Adenovirus**	1731	4.81	1712	98.90	19	1.10	Low
**Metapneumovirus**	1114	3.09	1104	99.10	10	0.90	Low
**Bocavirus**	362	1.01	358	98.90	4	1.10	Low
**Rhinovirus**	9710	26.97	9570	98.56	140	1.44	Moderate
**SARS-CoV-2**	2144	5.95	2091	97.53	53	2.47	High
**Another virus**	515	1.43	509	98.83	6	1.17	Moderate
**Total**	36,009	100.00	35,548	98.72	461	1.28	

Source: OpenDataSUS. * Respiratory Syncytial Virus (RSV).

**Table 4:** Final multivariate model for the risk of death in patients hospitalized for Severe Acute Respiratory Syndrome in children under 1 year of age in Brazil in 2024.

			95% Confidence Interval	
Variables	*p*-Value	OR *	Inferior	Superior
**Parainfluenza 4**	0.002	6806	2052	22,572
**Chronic kidney disease**	0.013	3.82	1334	10,936
**Immunodeficiency**	<0.001	3.68	1849	7322
**Down syndrome**	<0.001	3.59	2167	5.95
**Chronic heart disease**	<0.001	3129	2069	4731
**Chronic Neurological Disease**	0.005	2.25	1275	3969
**Oxygen Saturation < 95%**	<0.001	1758	1401	2205
**SARS-CoV-2**	0.005	1569	1144	2152
**Respiratory distress**	0.026	1.39	1041	1856
VSR **	0.005	0.736	0.595	0.909
**Cough**	<0.001	0.477	0.377	0.604
**Constant**	<0.001	0.013		

Source: OpenDataSUS. * Odds ratio, ** Respiratory syncytial virus. Model adjustments, Log-likelihood −2 (4071.566a), and Hosmer and Lemeshow test (*p*-0.693).

## Data Availability

Study data will only be available upon request to the corresponding author, PhD Daniele Sardinha danielle-vianna20@hotmail.com.
